# Spatial variability of the effect of air pollution on term birth weight: evaluating influential factors using Bayesian hierarchical models

**DOI:** 10.1186/s12940-016-0112-5

**Published:** 2016-02-05

**Authors:** Lianfa Li, Olivier Laurent, Jun Wu

**Affiliations:** 1 Program in Public Health, College of Health Sciences, University of California, Anteater Instruction & Research Bldg (AIRB) # 2034, 653 East Peltason Drive, Irvine, CA 92697-3957 USA; 2 State Key Lab of Resources and Environmental Information Systems, Institute of Geographical Sciences and Natural Resources Research, Chinese Academy of Sciences, A11 Datun Road, Anwai, Chaoyang, Beijing, 100101 China

**Keywords:** Bayesian hierarchical model, Spatial variability, Health effect, Air pollution, Term birth weight

## Abstract

**Background:**

Epidemiological studies suggest that air pollution is adversely associated with pregnancy outcomes. Such associations may be modified by spatially-varying factors including socio-demographic characteristics, land-use patterns and unaccounted exposures. Yet, few studies have systematically investigated the impact of these factors on spatial variability of the air pollution’s effects. This study aimed to examine spatial variability of the effects of air pollution on term birth weight across Census tracts and the influence of tract-level factors on such variability.

**Methods:**

We obtained over 900,000 birth records from 2001 to 2008 in Los Angeles County, California, USA. Air pollution exposure was modeled at individual level for nitrogen dioxide (NO_2_) and nitrogen oxides (NO_x_) using spatiotemporal models. Two-stage Bayesian hierarchical non-linear models were developed to (1) quantify the associations between air pollution exposure and term birth weight within each tract; and (2) examine the socio-demographic, land-use, and exposure-related factors contributing to the between-tract variability of the associations between air pollution and term birth weight.

**Results:**

Higher air pollution exposure was associated with lower term birth weight (average posterior effects: −14.7 (95 % CI: −19.8, −9.7) g per 10 ppb increment in NO_2_ and −6.9 (95 % CI: −12.9, −0.9) g per 10 ppb increment in NO_x_). The variation of the association across Census tracts was significantly influenced by the tract-level socio-demographic, exposure-related and land-use factors. Our models captured the complex non-linear relationship between these factors and the associations between air pollution and term birth weight: we observed the thresholds from which the influence of the tract-level factors was markedly exacerbated or attenuated. Exacerbating factors might reflect additional exposure to environmental insults or lower socio-economic status with higher vulnerability, whereas attenuating factors might indicate reduced exposure or higher socioeconomic status with lower vulnerability.

**Conclusions:**

Our Bayesian models effectively combined *a priori* knowledge with training data to infer the posterior association of air pollution with term birth weight and to evaluate the influence of the tract-level factors on spatial variability of such association. This study contributes new findings about non-linear influences of socio-demographic factors, land-use patterns, and unaccounted exposures on spatial variability of the effects of air pollution.

**Electronic supplementary material:**

The online version of this article (doi:10.1186/s12940-016-0112-5) contains supplementary material, which is available to authorized users.

## Background

Pregnant women and fetuses are vulnerable to adverse effects of air pollution [1–3]. Studies have associated air pollution exposures with adverse pregnancy outcomes [1, 4]. Exposure to toxic compounds in traffic-generated air pollutants may result in impaired placental hemodynamics with subsequent reduction of nutrients and oxygen supply, which reduces intrauterine growth and probably causes low birth weight [1]. The adverse health effect of air pollution is likely heterogeneous in space and possibly influenced by other environmental, socioeconomic, demographical and psychological factors [3, 5–7]. In particular, neighborhood socioeconomic status (SES) was found to be significantly associated with the heterogeneity of the effects of air pollution on birth weight [8, 9].

A few studies quantified between-region heterogeneity of air pollution effects. Dadvand et al. [10] reported stronger associations of reduction in term birth weight with higher median levels of particular matter (PM) with diameter <2.5 μm (PM_2.5_) across 14 study centers from North America, Europe, South America and Asia. Parker et al. [11] suggested that the composition of PM may influence the variability of the observed associations between PM mass and term birth weight in seven regions in the US. Williams et al. [12] quantified the spatially varying effects of sulfur dioxide and lead on birth weight across Census tracts in Tennessee. Recently, Coker et al. [13] and Hao et al. [14] respectively investigated the spatially varying effects of PM_2.5_ on low birth weight across Census tracts in Los Angeles and divisions in the contiguous United States. Most previous studies focused on particulate matter. Nitrogen dioxide (NO_2_) and nitrogen oxides (NO_x_) have been shown to be the best available indicators of local traffic emissions [15]. However, few studies have systematically investigated the spatial variability of the association between exposure to NO_2_ or NO_x_ and adverse pregnancy outcomes at a fine spatial resolution (e.g. Census tract).

As important geographic regions of survey and administration, Census tracts are designed to be relatively homogeneous with respect to population characteristics, socioeconomic status, and living conditions. On average, Census tracts has about 4000 (ranging from 1200 to 8000) inhabitants [16]. Socioeconomic status, demographics, and natural and built environment across Census tracts may modify the effects of air pollutants. In addition, spatial confounders may affect pregnancy outcomes, as shown in English et al. [17] where low birth weight was influenced by spatial autocorrelation. Confounders not captured in the models may result in biased estimates, which might lead to residual spatial autocorrelation (positive correlation between the residuals from the estimates made at nearby locations) [18]. Such unaccounted confounders can be partly accounted for by including spatial autocorrelation in the models [17, 19]. However, spatial autocorrelation has been ignored in many previous studies linking pregnancy outcomes to air pollution.

Previous studies have associated air pollution exposure with reduction in term birth weight [1, 3], although they reported varying effect sizes of air pollutants, possibly due to differences in study region, population, sample size, and exposure assessment methods [10]. We assume that the literature-reported mean effects of air pollution (weighted by the sample size) from independent studies follow a normal distribution according to the central limit theorem [20]. By using *a priori* knowledge of the effect of air pollution (i.e. quantitative summary of the effect sizes reported by previous studies), a Bayesian approach can be employed to combine *a priori* evidence and new data from a specific study setting to obtain the posterior estimates of the effects [21] in the study setting of interest.

Linear and logistic regressions have been used in most previous studies on the associations of air pollution and birth weight [1]. Logistic regression assumes a linear and additive relationship on a logistic scale, although the linear assumption may be over-simplistic for characterizing the influence of multiple factors [22, 23]. Non-linear methods have been used to directly evaluate associations between air pollution and birth weight [24, 25], and to adjust for individual confounding factors [26, 27]. In the non-linear methods, the penalized splines have been mostly used to construct the non-linear associations [28]; however, it may cause overfitting under the condition of a high number of degrees of freedom and a small size of sample. Bayesian hierarchical additive regression, while taking into account non-linear association and spatial effects, can combine *a priori* knowledge and new data to minimize potential overfitting.

This study aims to examine spatial variability of the associations between local traffic-related air pollutants (NO_2_ and NO_x_) and birth weight in term births (≥37 weeks) across Census tracts, and the influence of socio-demographic, land-use pattern and other spatial factors on these associations.

## Methods

**Fig. 1:**
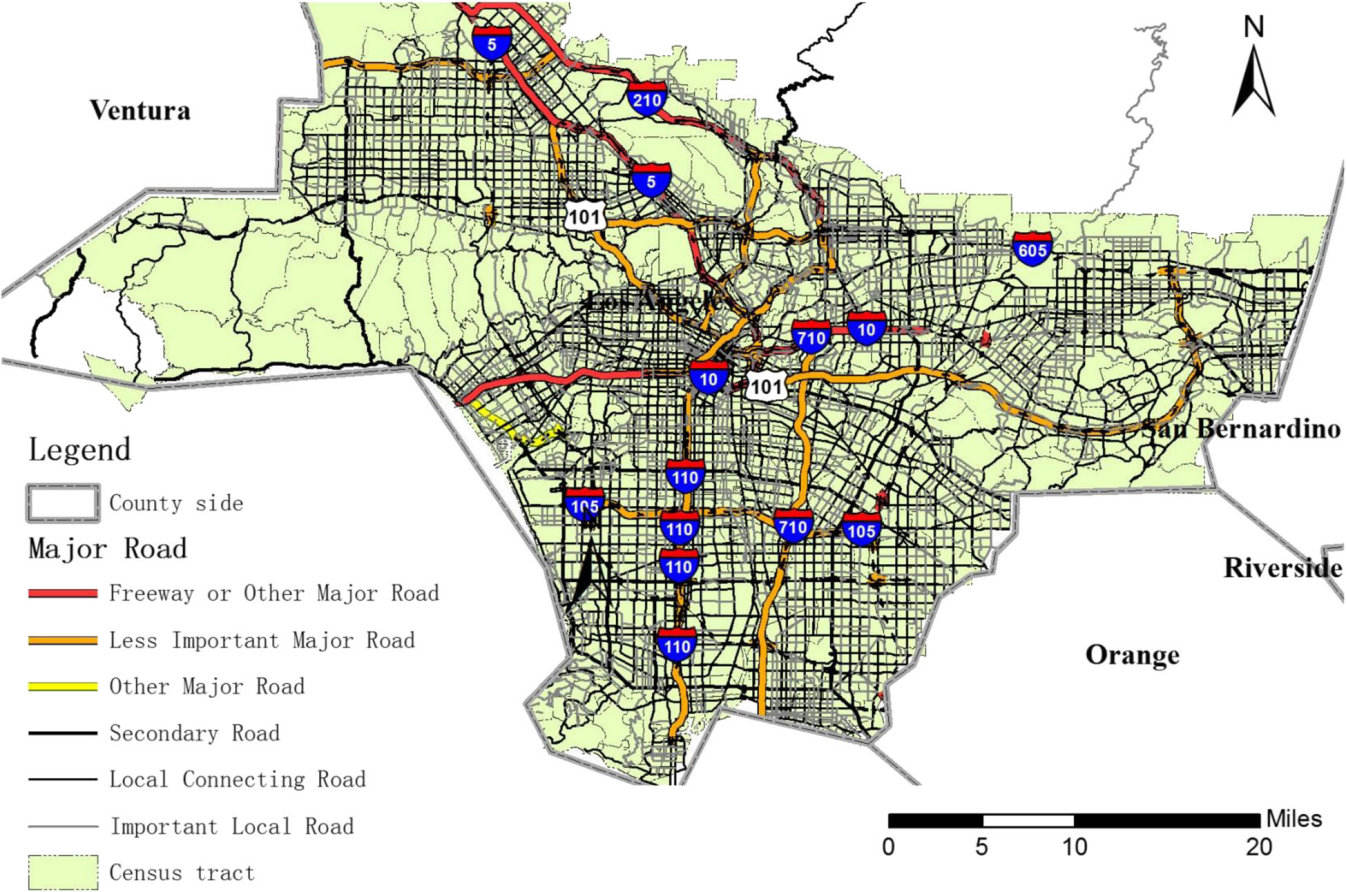
The Los Angeles Census tracts of this study. The black lines of one-dash-three-dots style indicate the boundaries for the Census tracts

This study domain covers Los Angeles County, California, USA. Birth certificate records from January 1, 2001 to December 31, 2008 (*n* = 1,203,782) were obtained from the California Department of Public Health. The birth data include birth weight, maternal residential address, and other individual-level variables (such as maternal age, length of gestation and primary health care). Maternal residential addresses on birth certificates were geocoded at the centroid of tax parcels whenever feasible using the University of Southern California Geographical Information System Research Laboratory geocoding engine [29]. The birth records were anonymized and de-identified prior to analysis for protection of privacy. Term birth was defined as births occurring between 37 and 45 weeks of gestation. Multiple births (*n* = 35,213) were excluded as well as infants with recorded birth defects (*n* = 3353) or unknown birth defects status (*n* = 398). The study domain included 2043 Census tracts (Fig. 1), among which 1948 tracts remained for analysis after we removed those with too large an area (>50 km^2^, approximately the top 98.3 % percentile by area; *N* = 37), too small sample size (less than 50 births; *N* = 29), islands (isolated Census tracts disconnected with any other tracts) (*N* = 12), or those with extreme values of term birth weight or covariates in the tracts (the outer fences [30] were used to filter the outlier tracts; *N* = 17). The 1.7 % very large tracts usually had a small population that were more heterogeneously distributed within the tracts than most of the other tracts, thus the statistics of environmental exposure and other parameters within the tract might not be representative of the whole tract. A very small sample size might introduce imprecision in model training, while the islands without neighbors would lead to difficulty in spatial modeling. Overall, we removed 95 (about 4.7 %) out of the 2043 tracts.

To study the association between air pollution and term birth weight, it is essential to obtain exposure estimates at a fine spatiotemporal resolution since air pollution can be highly heterogeneous in time and space [31, 32] and the fetus is likely to be more sensitive to air pollution during specific time windows of exposure [33, 34]. We employed an advanced spatiotemporal models recently developed and validated in the same study region [35] to estimate weekly NO_2_ and NO_x_ concentrations at the residence of each subject. Two major advantages of the models were the combination of long-term time-series data (high temporal but low spatial resolution) with dense sampling data from field campaigns (high spatial but low temporal resolution), and the incorporation of non-linear relationships between the predictor variables and the pollutant concentrations. The models showed good performance based on cross validation: for the weekly temporal trends, Pearson’s correlation was 0.84–0.91 for NO_2_ and 0.81–0.90 for NO_x_(Additional file 1: Figures S1 and S2). We averaged weekly exposure estimates to compute exposures in each of the three trimesters of pregnancy and the entire pregnancy period (more details in Additional file 1: Section 1).

According to *a priori* knowledge [1, 4] and the descriptive statistics of this study, we included the following confounding factors in the models: maternal age, length of gestation, ethnicity, educational level, parity, primary health care, and gender of the infant. In sensitivity analysis, we also tested the influence of pregnancy complications (i.e. diabetes, hypertension and preeclampsia) and their influence was limited. All the confounders were directly retrieved from the birth certificates.

Laurent et al. [36] showed the beneficial effect of greenness (indicated by normalized difference vegetation index [NDVI]) on birth weight for a subset of the present study’s population; thus, we included NDVI in this analysis as well. NDVI within a 500 m buffer of each residence was extracted and averaged based on a set of mostly cloud-free Landsat scenes (resolution: 30 m) from the Global Land Survey 2005 (United States Geological Survey) dataset covering Southern California.

Selection of the tract-level influential factors was based on *a priori* knowledge. We obtained information on these factors from the following sources:

1. Community survey data from the TIGER 2006–2010 5-year estimation [37]. The community survey data included the socio-demographic factors (median household income, percentages of race/ethnicity [Hispanic, White, Black or Asian], female educational level) as well as the factors that may be related to air pollution exposure at the tract level but were not accounted for in the individual-level exposure estimates (percentages of the people driving cars, trucks or vans to work, walking or bicycling to work, commuting time shorter than 30 min, using utility gas for heating). The commuting patterns of the population may directly influence personal exposures to traffic-related pollutants [38]. For these variables, we aggregated the Census block data to Census tracts by weighting the area of blocks within a tract.
2. Land-use patterns. We obtained the 2008 parcel-level land-use data from the Southern California Association of Government (SCAG). The percentages of areas for the following land-uses were extracted for each tract: low/high density residential community, heavy industry (including manufacturing, petroleum refining and processing, major metal and chemical processing and mineral extraction), electrical power facilities, park and recreational space (including local, developed or undeveloped parks and recreations, wildlife preserves and sanctuaries, specimen gardens and arboreta and beach parks).
3. Others: We obtained the 2005 TeleAtlas roadway network data from ArcGIS 10.1 (ESRI, Redlands, CA). The shortest distance to freeways/highways was calculated as the distance from the center point of each tract to the nearest freeways/highways. The same 30-m NDVI we used before for individual-level exposure was averaged over the entire area of each tract to characterize neighborhood greenspace.

Based on the Bayesian framework, we developed a hierarchical two-stage model to: 1) quantify the associations (effects) of individual air pollutants with term birth weight by adjusting for individual-level factors; and 2) investigate the influence of tract-level factors on the spatial variability of such effects.

Stage One: Within each Census tract, Bayesian additive model was used to link term birth weight with air pollution exposure while adjusting for individual-level confounding factors:

1 $$ \left\{\begin{array}{l}{y}_{ic}\sim N\left({\mu}_{ic},{\sigma}_c\right)\\ {}\mu \left({y}_{ic}\right)\  or\ \mathrm{t}\mathrm{r}\left(\mu \left({y}_{ic}\right)\right)={a}_{0c}+{x}_c^p{\beta}_c^p+{\displaystyle {\sum}_j{s}_c\left({x}_{jc}\right)+}{\displaystyle {\sum}_k{f}_c\left({x}_{kc}\right)+{\varepsilon}_c}\\ {}\mu \left({y}_{ic}\right)=E\left({y}_{ic}\Big| All\ \mu \left({y}_{lc\left(l\ne i\right)}\right)\right)\end{array}\right. $$

where *c* is the index of Census tract (*c* = 1,…,*n*), *y*_*ic*_ is the *i*^th^ individual term birth weight for the tract *c*, *μ*(*y*_*ic*_) is the expected value of the target variable (*y*_*ic*_), tr(*μ*(*y*_*ic*_)) is the transformation (e.g. log, box-cox) of *μ*(*y*_*ic*_), *x*_*c*_^*p*^ is the average of the *p*^th^ air pollutant (NO_2_ or NO_x_) during a study period, *a*_0*c*_ is the intercept, *β*_*c*_^*p*^ is the regular health effect (change in term birth weight per unit increase in exposure) of the *p*^th^ air pollutant; *x*_*jc*_ indicates the *j*^th^ continuous confounder such as NDVI and maternal age, while *x*_*kc*_indicates the factor variables such as race/ethnicity, parity and educational level. ***s***_***c***_() is a semi-parametric spline function and *f*_*c*_() is a factor function. *y*_*ic*_, *a*_0*c*_ and *β*_*c*_^*p*^ are assumed to be normally distributed: *y*_*ic*_ ~ *N*(*μ*_*c*_, *σ*_*c*_), *a*_0*c*_ or *β*_*c*_^*p*^ ~ *N*(0, *σ*_*p*_). *μ*(*y*_*ic*_) is the expected value of the *i*^th^ individual *y*_*ic*_ conditional on their neighborhood [*E*(*y*_*ic*_|*All μ*(*y*_*lc*(*l* ≠ *i*)_))] and modeled using spatial residuals, *ε*^*c*^ ~ *N*(0, *Σ*^*c*^). *Σ*^*c*^ = [*σ*_*ij*_^*c*^] represents spatial autocorrelation (*σ*_*ij*_^*c*^ based on the distance between the *i*^th^ and *j*^th^ subject locations, modeled using the variogram). We used Moran’s I [39] to determine the magnitude of spatial autocorrelation and whether it is necessary to incorporate it into the model [40]. In the end, we did not include spatial autocorrelation in the stage one model since insignificant spatial autocorrelation was found for 91 % of the Census tracts.

The associations of air pollution with term birth weight were estimated based on a posterior distribution using full Bayesian inference via Markov Chain Monte Carlo (MCMC) simulation, which updated full conditionals of single or blocks of parameters [41]. Additional file 1: Section 2 presents the details for this.

**Fig. 2:**
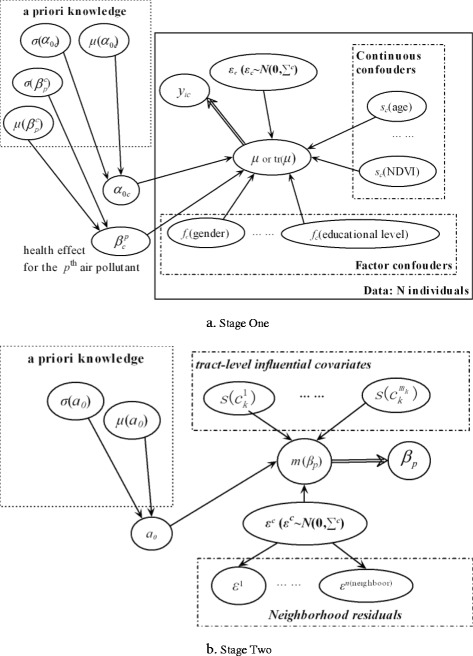
Two-stages Bayesian modeling framework **a**. Stage One; **b**. Stage Two. The circles or ellipses represent the random variables; the arrow lines indicate the influential relationship (association) from the staring node to the ending node of the line

Figure 2a shows the stage one model of our approach, in which the intercept (*a*_0*c*_) and effect coefficient of air pollutant (*β*_*p*_^*c*^) were assumed to be normally distributed and their hyper-parameters (mean: *m*; standard variance: *σ*) were derived from *a priori* knowledge based on the systematic summarized findings from the literature on the effects of NO_2_ on term birth weights after weighting by the sample size (Additional file 1: Table S1). To avoid double counting, we excluded the meta-analysis papers and only included the individual studies that they covered (plus other independent studies) without duplicates in the summary (Additional file 1: Figure S3). We also made sensitivity analyses using the pooled estimates reported by Stieb et al. [3] (−14.1 g per 10 ppb for NO_2_) as *a priori* knowledge in the models.

Only a few published studies reported the association between NO_x_ and term birth weight and mixed results were observed [42]. Therefore, we assumed a uniform distribution of the NO_x_ effect with a mean of 0 (no effect) with a standard deviation of 1 as non-informative prior knowledge [3, 43].

Stage Two: the effects (*β*) of air pollution on term birth weight were modeled against the tract-level covariates to examine their influence on spatial variability of *β*.

2 $$ \left\{\begin{array}{l}{\beta}_p\sim {N}_k\left(m\left({\beta}_p\right),{V}_p\right)\\ {}\mu \left({\beta}_p\right)=h\left({\eta}_p\right)\\ {}{\eta}_p={\alpha}_0+{\displaystyle {\sum}_{j=1}^{m_p}s\left({c}_j\right)+{\varepsilon}_p}\\ {}\mu \left({\beta}_p\right)=E\left({\beta}_p\Big|Nei\left({\beta}_{pj\left(j\ne c\right)}\right)\right)\end{array}\right. $$

where *p* represents the *p*^th^ air pollution exposure, *μ*(*β*_*p*_) represents the expected estimates for *β*_*p*_, *h*(*η*_*p*_) is the link function for *μ*(*β*_*p*_) (*h*(*η*_*p*_) = *η*_*p*_ for normal distribution), *c*_*j*_ are the influential factors at the tract level, *s*(*c*_*j*_) is the semi-parametric non-linear spline function for the factor *c*_*j*_. The intercept, *α*_0_ represents the average estimate of air pollution effect [*α*_0_ ~ *N*(0, *σ*_*p*_)]. *ε*_*p*_ is assumed to be spatially auto-correlative (*ε*_*p*_|*Σ* ~ *N*_*p*_(0, *Σ*^*p*^)) and is modeled by spatially conditional auto-correlative regression (∑^*p*^ = [*σ*_*ij*_^*p*^] represents spatial covariance) [44]. The variance of *β*_*p*_ measures the variability of the association between air pollution and term birth weight across Census tracts. Moran’s I was used to determine whether spatial autocorrelation of the effects of air pollution should be included in the stage two model.

The conditional expectation of the target variable (*β*_*p*_) incorporates spatial effects [41] and is determined by Census tract-level covariates and the weighted sum of the residuals of the effect coefficient from the means at neighborhoods [*Nei*(*β*_*pj*(*j* ≠ *c*)_) in Eq. (2)]. In this study, spatial adjacency is based on the rook type that defines two tracts with at least one shared common boundary as neighbors [45]. The residual to incorporate spatial influence from neighborhood is:

3 $$ {\varepsilon}_p=\rho {\displaystyle {\sum}_{j\ne c}{w}_{cj}\left({\beta}_{pj}-m\left({\beta}_{pj}\right)\right)} $$

where *ρ* represents the effects of adjacent neighbors to be estimated, *w*_*cj*_ are spatial weights determining the relative influence of neighborhood Census tract *j* on Census tract *c* [44] (*w*_*cj*_ = 1 if tract *c* is an adjacent neighbor of tract *j*; *w*_*cj*_ = 0 otherwise).

Point estimates (means) of the posterior effects of air pollutants and the impact of tract-level factors on the effects were calculated based on the posterior distribution using full Bayesian inference in the stage two model, which is similar to Bayesian inference via Markov Chain Monte Carlo simulation in stage one. Additional file 1: Section 2 provides more details for the models. In the stage one and two models, we ran nine times of MCMC simulations and used the Gelman and Rubin approach to diagnose the convergence of the simulations to the stationary posterior distribution [45].

Figure 2b shows the stage two model of the Bayesian framework. The hyper-parameters (mean: *μ*; standard variance: *σ*) of the tract-level influential factors were determined according to *a priori* knowledge or as uninformative priors. The outputs included the uncertainty of the posterior estimates of the effects, and the 95 % credible intervals (CI).

For evaluation of Bayesian hierarchical models, we used deviance information criterion (DIC) as a generalization of the Akaike information criterion and Bayesian information criterion. In Bayesian models, DIC has the advantage over other criteria mainly because it can be easily calculated from the samples generated by a Markov Chain Monte Carlo simulation. Smaller values of DIC indicate a better fitting model.

Although some variables (e.g. ethnicity and NDVI) were simultaneously used in the stage one and two models, they represented individual-level characteristics in the stage one model, and neighborhood or context characteristics as aggregated features in the stage two model [46, 47]. We treated ethnicity as a categorical variable in stage one, but as continuous variables (e.g. percentages of Hispanic, White, Black and Asian) in stage two. The NDVI was extracted as the average over a 500 m buffer of a specific residence in stage one but as the average over the entire Census tract in stage two.

Our models were constructed in R 3.2.1 (Bell Laboratories, New Jersey, US) with the JAGS [48] and BayesX [49] packages. Details about the use of the packages are described in the last paragraph in Additional file 1: Section 2. We also include the main codes used for the two stage models as Additional files 2, 3, 4 and 5.

The study has been approved by the Institutional Review Board of the University of California, Irvine.

## Results

Table 1 presents summary statistics (mean, median and inter-quartile range [IQR]) for the means of individual-level variables across 1948 Census tracts (statistics of Census tracts: mean area: 4.0 km^2^; variance of the areas: 26.9 km^2^). Mean term birth weight was 3393 g with an IQR of 58 g among all the tracts. Spatiotemporally-modeled NO_2_ and NO_x_ exposures (IQR: 11.4 ppb for NO_2_; 31.3 ppb for NO_x_) had high variability (defined as standard variance divided by mean: 0.42 for NO_2_, 0.83 for NO_x_). Table 2 lists the statistics of the association between air pollution and term birth weight for each individual tract across all the tracts. Since a stronger association was found for exposure during the entire pregnancy than trimester-specific exposures (Table 2), we mainly reported the results for exposure during the entire pregnancy period. The DIC value was 454 for NO_2_ and 465 for NO_x_ and the difference in DIC was relatively small (Table 2).

**Table 1:** Statistics for the means of the target and individual-level variable across Census tracts

Type	Variable	Category	Mean	Median	Inter quartile range (IQR)^a^
Target variable	Term birth weight (g)		3393	3392	58 (3363,3421)
Pollution indicator	Spatiotemporal NO_2_ (ppb)^b^		25.00	24.14	11.43 (18.74, 30.17)
	Spatiotemporal NO_x_ (ppb)^c^		17.32	26.31	31.32 (15.48, 46.80)
Individual- level variable	NDVI		0.26	0.25	0.09 (0.21, 0.30)
	Maternal age		28.8	28.1	4.1 (26.7, 30.8)
	Length of gestation		277	276	12 (271,283)
	Infant gender (percentage)	Male	49 %	49 %	6 % (46–52 %)
		Female	51 %	51 %	6 % (48–54 %)
	Race/ethnicity (percentage)	Hispanic	54 %	57 %	57 % (26–83 %)
		White	24 %	12 %	43 % (2–45 %)
		Black	6 %	3 %	5 % (0.8–6 %)
		Asian	13 %	8 %	14 % (2–16 %)
		Others	2 %	1 %	2 % (1–3 %)
	Maternal educational level (percentage)	Less than 8th grade	10 %	8 %	15 % (1–16 %)
		9th grade to high school	42 %	47 %	36 % (24–60 %)
		Less than 4 years of college	29 %	20 %	43 % (6–49 %)
		4+ of college	19 %	19 %	11 % (13–24 %)

^a^IQR for the mean (continuous variables) or percentage (categorical variables);

^b^Estimates of NO_2_ by the spatiotemporal model; ^c^Estimates of NO_x_ by the spatiotemporal model

**Table 2:** Statistics of the effects^a^ of NO_2_ and NO_x_ on term birth weight across all the tracts

Pollutants	Period	Mean (g/ppb)	95 % confidence intervals	Mean of deviance information criterion
NO_2_(ppb)	1st trimester	−0.99	[−1.39, −0.59]	512
	2st trimester	−0.79	[−1.26, −0.32]	501
	3st trimester	−1.27	[−1.72, −0.82]	505
	Entire Pregnancy	−1.89	[−2.23, −1.55]	454
NO_x_(ppb)	1st trimester	−0.54	[−0.80, −0.28]	514
	2st trimester	−0.52	[−0.78, −0.26]	512
	3st trimester	−0.61	[−0.90, −0.32]	512
	Entire Pregnancy	−0.87	[−1.09, −0.65]	465

^a^Change in term birth weight (g) per unit increase in exposure to air pollution (ppb)

From the existing literature, the summary of the prior effects of air pollution on term birth weight confirmed the normal distribution of the effect size (Additional file 1: Figure S3). Our sensitivity analysis showed limited influence of *a priori* knowledge on posterior estimates (means NO_2_ effect: −13.7 g per 10 ppb [our summary] vs. −13.9 g per 10 ppb [Stieb’s pooled estimate]) despite the small to moderate differences in *a priori* estimates using our summary (as *a priori* knowledge, Additional file 1: Table S1 and Figure S3) vs. the pooled estimates [3].

Global Moran’s I tests showed spatially-clustering distribution of the associations between air pollutants and term birth weight. The Z-scores (6.4 for NO_2_; 6.1 for NO_x_) were outside the range of −2.5 and 5.4 with *p-value* < 0.01, indicating moderate to strong spatial autocorrelation [50]. Therefore, we incorporated spatial effects in stage two.

The potential scale reduction factor for the nine MCMC simulations ranged from 1.01 to 1.03, approaching 1.0, indicating a stationary convergence to the posterior distribution. The overall intercept as the tract-level posterior average effects of air pollutants on term birth weight (Additional file 1: Table S3) was −14.7 (95 % CI: −19.8, −9.7) g per 10 ppb for NO_2_, and −6.9 (95 % CI: −12.9, −0.9) g per 10 ppb for NO_x_.

**Fig. 3:**
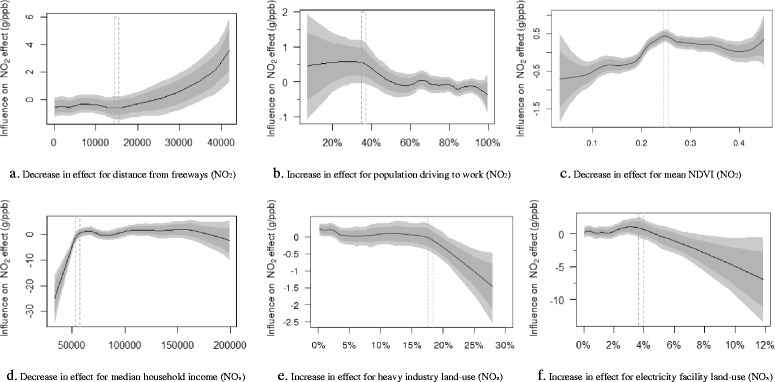
Non-linear influence of the tract-level factors on effects of air pollutants on term birth weight. The gray dash lines indicate the approximate intervals of thresholds where the influential factors start to take pronouncedly attenuating (**a**, **c** and **d**) or aggravating (**b**, **e**, **f**) influence on effects of air pollutants. The shades around the curve indicate the 95 % pointwise confidence limits of the estimate acquired by the hierarchical models

We found significant non-linear associations of exposure-related factors, socio-demographic factors, land-use patterns, and greenness with spatial variability of air pollution effects across Census tracts; the non-linear trends varied by these factors. The results of linear models (Additional file 1: Table S2) obscured significant nonlinear trends of such associations. The non-linear model captured the thresholds of the influential factors when their effects started to change markedly (Fig. 3 and Additional file 1: Figures S4 and S5). For illustration purposes, Additional file 1: Table S3 shows the changes in the average effects of air pollutants between the 1st and the 4th quartiles of the tract-level factors, compared with the average posterior effects as the reference.

We found that the tracts further away from freeways/highways generally had smaller reduction in term birth weights associated with exposure to NO_2_ and NO_x_ (less weight reduction from the 1st to the 4th quartile of the distance: 3.7 g per 10 ppb for NO_2_; 4.9 g per 10 ppb for NO_x_). The tracts with a higher percentage of the people driving to work (approximately 36–60 %) were associated with higher reduction in term birth weight for exposure to NO_2_ and NO_x_ (more weight reduction from the 1st to the 4th quartile of the percentage of the population driving to work: 1.6 g per 10 ppb for NO_2_; 14.7 g per 10 ppb for NO_x_). Additionally, the tracts with more people using gas for indoor heating had larger reduction in term birth weight from air pollution (more weight reduction from the 1st to the 4th quartile of the percentage of the population using gas for indoor heating: 1.8 g per 10 ppb for NO_2_; 17.4 g per 10 ppb for NO_x_).

For the tract-level socio-demographic factors, household income, ethnicity and education level played important roles in spatial variability of the health effects on term birth weight. Higher household income was associated with smaller reduction in birth weight from air pollution exposure (less weight reduction from the 1st to the 4th quartile: 1 g per 10 ppb for NO_2_; 3.4 g per 10 ppb for NO_x_). Race/ethnicity showed significant influence on the effects of air pollution: a higher percentage of the Whites were associated with a lower risk of reduced birth weight from air pollution exposure (less weight reduction from the 1st to the 4th quartile: 0.3 g per 10 ppb for NO_2_). Further, a higher percentage (>10 %) of the women with no or low education (below bachelor level) was associated with a higher risk of reduced birth weight from NO_2_ exposure (more weight reduction by 5.1 g per 10 ppb from the 1st to the 4th quartile).

For the land-use patterns, a higher percentage (>approximately 18 %) of heavy-industry land-use (range: 0–30 %) was linked to larger effects of NO_2_ and NO_x_ on reduced birth weight (more weight reduction by 13.0 g per 10 ppb of NO_2_ and 3.9 g per 10 ppb of NO_x_, about 56–88 % of the tract-level posterior average effects, from the 1st to the 4th quartile of the percentage of heavy-industry land-use area]. A higher percentage (>5.8 % for NO_2_; >3.8 % for NO_x_) of electrical power facilities land-use (range: 0–12 %) resulted in higher risks of NO_2_ and NO_x_ (more weight reduction by 31.4–62.3 g per 10 ppb from the 1st to the 4th quartile). Conversely, park and recreational land-use and NDVI (Fig. 3-f) were respectively associated with smaller reduction in birth weight from NO_x_ (less weight reduction by 6.7 g per 10 ppb from the 1st to the 4th quartile) and NO_2_ (less weight reduction by 4.8 g per 10 ppb from the 1st to the 4th quartile) exposure.

Spatial effects accounted for an important proportion (35–46 %) of the spatial variability of air pollution effect on term birth weight. Additional file 1: Figure S6 showed moderate-to-strong spatial clustering of the spatial distribution of posterior effects of NO_2_ (a) and NO_x_ (b) on term birth weight and the spatial clustering patterns were similar between the two pollutants. Additional file 1: Figure S7 presents the uncertainty of the results, i.e. the probability that air pollution effect is negative (adverse effect) in a given Census tract.

## Discussion

Using the two-stage hierarchical Bayesian models, we quantified the effects of air pollution exposure on term birth weight and examined Census tract-level factors contributing to the spatial variability of such effects. Overall, the posterior estimates confirmed the negative association between air pollution exposure and term birth weight although the associations with NO_2_ were more supported by the previous studies than that with NO_x_. The sensitivity analysis shows small difference between the prior effects of NO_2_ and our posterior estimates. This may reflect the consistency about the adverse effect of NO_2_ on term birth weight. The average posterior output showed adverse effects of NO_2_ and NO_x_ on term birth weight: NO_2_ and NOx may suppress antioxidant defense system, cause lipid peroxidation and disturb fetus development, thus potentially leading to low birth weight [2].

To our knowledge, this is the first study employing the Bayesian non-linear approach to examine spatial variability of the effects of air pollution on term birth weight across Census tracts and the factors contributing to such variability. Whereas the previous studies used the penalized smooth splines to adjust for the covariates [26, 27] or to simulate non-linear exposure-response associations [24, 25], this study used Bayesian hierarchical regression to quantify non-linear trends for the influence of the Census tract-level factors on the associations between ambient air pollutant concentrations and term birth weight, e.g. identifying the thresholds where the influence had significant marked change, as shown in Fig. 3. Compared to the other non-linear methods, the Bayesian approach can minimize over-fitting by combining data with *a priori* knowledge; the latter is used as a penalty on the parameters (e.g. effects of NO_2_ on term birth weight) to be learned to reflect the prior knowledge. Epidemiological studies have frequently linked air pollution with adverse birth outcomes [3]. Based on the literature, we summarized the mean effect and the variance, and use them as *a priori* knowledge in the Bayesian models. The variances of *a priori* knowledge and the training sample determine the weights between both [43]: if the prior mean effect has a small variance, indicating relatively consistent findings from the previous studies, higher weight is assigned to the prior mean effect; conversely, if the prior mean effect has a high variance, indicating heterogeneity in previous findings, lower weight is given to the prior mean effect and higher weight is given to the training sample. Thereby, the use of variances as the weights for *a priori* knowledge and data can effectively control the influence of over-smoothing by using the prior mean. Further, the incorporation of spatial effects within the Bayesian model enabled the identification of spatial patterns of the adverse effects of NO_2_ and NO_x_ on term birth weight.

The emissions of air pollutants not accounted for by NO_2_ and NO_x_ exposures may influence the spatial variability of the effects of the two pollutants on term birth weight. Being away from freeway/highway might lead to lower local traffic-related air pollutant exposure and lower noise exposure at residential locations, thus lowering the effect of NO_2_ and NO_x_ at the Census tract level. We found lower risk of air pollution for reduction in term birth weight in Census tracts with a higher percentage of people biking or walking to work, or with a higher percentage of people who commute less than 30 min to work. For the working population taking vehicles, average exposure to traffic-related pollutants may be higher due to their exposure in commutes [38]. Further, more work-related commutes may increase the concentrations of traffic-related air pollution in local communities that were not captured by the spatiotemporal models for NO_2_ and NO_x_. Additionally, long commuting time is likely associated with a higher stress level (e.g. driving activity itself and shorter time with family) that might lead to the observed higher risk associated with air pollution [8, 51].

Besides ambient pollution, we observed that house heating was significantly associated with increase of the tract-level adverse effects. This may indicate an effect of increased exposure to indoor air pollution [52]. Although house heating is uncommon in Los Angeles, the availability of gas heaters may indicate a 50 % probability of use of gas stove at least for cooking according to the US Residential Energy Consumption Survey [53, 54]. Gas stove use may increase indoor concentrations of NO_2_, particulate matter and other pollutants [52, 55, 56], which may add to the observed risk of NO_2_ and NO_x_.

This study suggests important influence of socio-demographic factors. Higher household income reflects higher SES and likely lower vulnerability to the effects of air pollution, consistent with the previous studies [9, 57]. Results also showed a non-linear trend: beyond the threshold of about $40,000–60,000, the influence of income on the effects of air pollution became much weaker [58].

Further, Census tracts with a higher percentage of Whites had a lower risk of reduced birth weight. Previous studies [6, 57, 59] consistently showed weaker adverse effects of air pollution in White mothers than in African American mothers. Besides genetic differences, SES in different race groups may also contribute to the observed differences in health effects. In our study population, tract-level median household income was negatively associated with the percentage of African Americans and positively correlated with the percentage of Whites (Pearson’s correlation: −0.19 vs. 0.54).

Higher maternal education may indicate higher SES, more knowledge on potential adverse effects of environmental agents, and healthier life style, which may reduce the adverse effects of air pollution on pregnancy outcomes. Our results agree with a number of previous studies that linked lower maternal educational level with adverse pregnancy outcomes including term low birth weight [6].

Land-use patterns may indicate more or less exposures to uncounted air pollution sources or to green space. Typically, heavy industry might produce higher local emissions of air pollution or other pollutants not captured by our exposure measures and models. In fact, industry land-use was not included as a predictor in our spatiotemporal models. Heavy industry might also be associated with other environmental insults such as noise. Further, we found a non-linear trend, with a threshold (approximately 18 % of area) for heavy industrial land-use, above which the influence was noticeably exacerbated for the effects of NO_2_ and NO_x_. Further, the land-use of electrical power facilities had significant influence on the effects. Electrical power facilities included electric power installation, welding, induction heaters and electrified transport systems that were important sources of extremely low frequency fields [60]. We found non-linearly increased adverse effects of air pollution on term birth weight beyond the threshold of 3.8–5.8 % of area for electrical power facilities. Several studies [61–63] showed adverse influence of the extremely low frequency fields on pregnant women. A possible mechanism for this is that the extremely low frequency fields might disturb the balance between plasma and vascular cell Ca^2+^, subsequently resulting in disruption of placental vascular function change and suboptimal growth of the fetus, thus potentially impairing fetal growth. However, more investigation is required to evaluate whether the electrical power facility land-use as a neighborhood factor might aggravate the adverse effects of air pollution.

Contrarily, the exposure to the park and recreational land-use and greenness was associated with a smaller reduction in term birth weight from NO_2_ and NO_x_ exposure. A higher value of the two variables might be associated with more active social and physical activities in the neighborhoods [64], reduced local temperature and exposure level to air pollution and noise, and less stress [65]. Our findings on the beneficial effects of greenness are consistent with the previous studies [36, 64, 66].

Traditional regression analysis, without considering spatial effects, may generate the estimates with spatially auto-correlative residuals [67]. In this study, NO_2_ and NO_x_ had similar patterns of spatial clustering. Strong spatial patterns might indicate significant influence of the regional factors such as regional patterns (potentially correlated with pregnancy outcomes) in diet and lifestyle, and surrounding terrain or physical setting (not included in the model) on the health effects of air pollution.

Our finding of spatially varying effects of air pollution is consistent with the recent finding of Coker et al. [13] for the same study region (Los Angeles). Our study advanced the previous studies [12–14] by using Bayesian hierarchical models to assess which factors contributed to the varying spatial effects of air pollution. We identified the tract-level factors that attenuate or exacerbate the association between air pollutants and term birth weight.

This study has several limitations. First, *a priori* effect of air pollution on term birth weight was derived from a limited number of studies that either used air pollution measures of relatively coarse spatial resolution or used simplistic regression models. To minimize the potential inconsistency and bias in the estimation of air pollution effects, we incorporated the variance of the prior evidence as an uncertainty indicator and used it as weights between data learning and *a priori* knowledge. Second, although advanced spatiotemporal models were used to estimate air pollutant concentrations, there was still uncertainty in estimating the personal exposure of pregnant women. We did not consider exposures at workplace and in vehicles. In addition, we relied on the address at delivery for exposure assessment and did not consider the change of address during pregnancy due to a lack of data. This might introduce exposure misclassification for a limited number of mothers. Third, we did not account for the influence of multiple pollutants. However, it is difficult to put the pollutants together in one model since this may introduce co-linearity (we found strong correlation [Pearson’s r = 0.81] between NO_2_ and NO_x_). Fourth, although our non-linear models detected the thresholds of the land-use from which the influences on the effects of air pollutants had pronounced variations, such thresholds may vary across cities or regions. For example, the heavy-industry land-use may be affected by the type of industry, spatial distribution of population and emission sources, and thus such threshold may vary by city. In addition, the greenness indicator of NDVI did not specify types of vegetation, which may differ by region and have different influence on the effects of air pollutants. However, the spatial effect included in our models might partially account for such confounding effects. Fifth, our approach quantified the associations between the influential factors and the effects of air pollutants, but such associations were not necessarily causal. For example, the distance from highway may just represent suburban tracts, which are very different from the urban tracts.

## Conclusions

This study developed a new two-stage hierarchical model based on a Bayesian framework to quantify the effects of air pollution exposure on term birth weight and examine the factors contributing to spatial variability of such effects across Census tracts. The posterior results confirmed the adverse effect of air pollution on term birth weight. The effects of air pollution varied across Census tracts, and were significantly influenced by the tract-level exposure-related environmental, socio-demographic and land-use factors. We detected non-linear thresholds from which the influential factors started to pronouncedly attenuate or exacerbate the influence of air pollutants. Further, modeled spatial effect accounted for a large portion of the variance explained. Our results can inform the public and the decision makers about the spatial distribution of the health effects of air pollution across Census tracts and the influential factors of such effects.

## Additional files

Additional file 1: **Section 1.** Spatiotemporal models for exposure estimation of NO_2_ and NO_x_. **Section 2.** Two-stage models. **Table S1.** Effects of NO_2_ and NO_x_ on birth weight from the previous studies. **Table S2.** Contribution of each tract-level factor in linear models to the NO_2_ and NO_x_ effects. **Table S3.** Change in effects of NO_2_ and NO_x_between the 1st and 4th quartiles of each tract-level factor in non-linear models. **Figure S1.** NO_2_ and NO_x_ time series predicted by the spatiotemporal model. **Figure S2.** NO_2_ (a) and NO_x_ (b) long-term averages of the predicted time series vs. the measurements for all the 25 stations. **Figure S3.** *A priori* statistics of the effects of NO_2_ on birth weight summarized from the previous studies (Table S1). **Figure S4.** Non-linear effects of NO_2_ on birth weight (smooth term) from Stage Two. **Figure S5.** Non-linear effects of NO_x_ on birth weight (smooth term) from Stage Two. **Figure S6.** Posterior estimates of the effects of NO_2_ and NO_x_. **Figure S7.** Probability map of the Census tract NO_2_ and NO_x_ effects for term birth weight [P(β < 0)]. (PDF 1852 kb)

Additional file 2: **Stage one no2: Code file for the stage one model for NO**_2_. (R 6 kb)

Additional file 3: **Stage one nox: Code file for the stage one model for NO**_x_. (R 5 kb)

Additional file 4: **Stage two no2: Code file for the stage two model for NO**_2_. (R 5 kb)

Additional file 5: **Stage two nox: Code file for the stage two model for NO**_x_. (R 5 kb)

## Abbreviations

CI: Credible intervals
DIC: Deviance information criterion
IQR: inter-quartile range
MCMC: Markov Chain Monte Carlo
NDVI: Normalized difference vegetation index
NO_2_: Nitrogen dioxide
NO_x_: Nitrogen oxides
PM: Particular matter
PM_2.5_: Particular matter with diameter <2.5 μm
SCAG: Southern California Association of Government
SES: Socioeconomic status
