# Supplementary material for: Spatial variability of the effect of air pollution on term birth weight: evaluating influential factors using Bayesian hierarchical models
Source: Environ Health. 2016 Feb 5;15:14. doi: 10.1186/s12940-016-0112-5 (PMC4744429; doi:10.1186/s12940-016-0112-5)
Supplement: Supplementary file 1 — Section 1. Spatiotemporal models for exposure estimation of NO2 and NOx. Section 2. Two-stage models. Table S1. Effects of NO2 and NOx on birth weight from the previous studies. Table S2. Contribution of each tract-level factor in linear models to the NO2 and NOx effects. Table S3. Change in effects of NO2 and NOxbetween the 1st and 4th quartiles of each tract-level factor in non-linear models. Figure S1. NO2 and NOx time series predicted by the spatiotemporal model. Figure S2. NO2 (a) and NOx (b) long-term averages of the predicted time series vs. the measurements for all the 25 stations. Figure S3. A priori statistics of the effects of NO2 on birth weight summarized from the previous studies (Table S1). Figure S4. Non-linear effects of NO2 on birth weight (smooth term) from Stage Two. Figure S5. Non-linear effects of NOx on birth weight (smooth term) from Stage Two. Figure S6. Posterior estimates of the effects of NO2 and NOx. Figure S7. Probability map of the Census tract NO2 and NOx effects for term birth weight [P(β < 0)]. (PDF 1852 kb) [file 12940_2016_112_MOESM1_ESM.pdf]

Additional file 1: Supporting Information

Spatial Variability of the Effect of Air Pollution on Term Birth Weight: Evaluating Influential Factors Using Bayesian Hierarchical Models

Lianfa Li[1, 2], Olivier Laurent, Jun Wu[1]

1 Program in Public Health, College of Health Sciences, University of California, Irvine

2 State Key Lab of Resources and Environmental Information Systems, Institute of Geographical Sciences and Natural Resources Research, Chinese Academy of Sciences

Contents

1. Spatiotemporal models for exposure estimation of NO_2 and NO_x…………………1

2. Two-stage models …………………………………………………………………………1

Table S1. Effects of NO_2 and NO_x on birth weight from the previous studies……………..5

Table S2. Contribution of each tract-level factor in linear models to the NO_2 and NO_x effects……………………………………………………………………………………..6

Table S3. Change in effects of NO_2 and NO_x between 1st and 4th quartiles of each tract-level factor in non-linear models………………………………………..……..…....7

Figure S1. NO_2 and NO_x time series predicted by the spatiotemporal model……..……………………………………………………………………………........8

Figure S2. NO_2 (a) and NO_x (b) long-term averages of the predicted time series vs. the measurements for all the 25 stations………………………………………………………..9

Figure S3. *A priori* statistics of the effects of NO_2 on birth weight summarized from the previous studies …………………………………………………………………………...10

Figure S4. Non-linear effects of NO_2 on birth weight (smooth term) from Stage Two ……………………………………………………………………………..…. 11

Figure S5. Non-linear effects of NO_x on birth weight (smooth term) from Stage Two……………………………………………………………………………........…13

Figure S6. Posterior estimates of the effects of NO_2 and NO_x…………………………....15

Figure S7. Probability map of Census tract NO_2 and NO_x effects for term birth weight [$P(\beta < 0)$]……………………..……………………………………………………………16

References……………..………………………………………………………………………17

1. Spatiotemporal models for exposure estimation of NO_2 and NO_x

For this study, this spatiotemporal model was learned from existing NO_2 and NO_x measurements (averaged weekly) from different sources, including routine measurements from the South Coast Air Quality Management District (SCAQMD, time series of 10 years from 2000-2009), and episodic measurements from University of California Irvine (UCI, four weekly measurements in 2009) and University of California Los Angeles (UCLA, two bi-weekly measurements respectively in 2006 and 2007). Then, the weekly concentrations of NO_2 and NO_x were estimated and averaged respectively for each of the three trimesters and the entire pregnancy period at each subject locations.

This trained model had a good cross-validation performance: (1) for the time trends, Person's correlation was 0.84-0.91 for NO_2 and 0.81-0.90 for NO_x (Figure S1 of Additional file 1); (2) for the long-term averages at the 25 SAQMD locations, R^2 was 0.95 for NO2 and 0.73 for NO_x (Figure S2 of Additional file 1).

2. Two-stage models

This section shows the details of the two-stage approach to examine spatial variability of the effects of air pollution across Census tracts, and influence of the exposure-related, socio-demographic, land-use pattern and greenness factors at the tract level on it.

Stage One: Within each census tract, the association between air pollution exposure and term birth weight was established using the Bayesian additive model which takes into account potentially confounding factors at individual level:

$$\begin{cases} y_{ic} \sim N(\mu_{ic}, \sigma_c) \\ \mu(y_{ic}) \text{ or } \mathrm{tr}(\mu(y_{ic})) = a_{0c} + x_c^p \beta_c^p + \sum_j s_c(x_{jc}) + \sum_k f_c(x_{kc}) + \varepsilon_c \\ \mu(y_{ic}) = E(y_{ic} \mid \text{All } \mu(y_{lc(l \neq i)})) \end{cases} \quad (1)$$

where c is the index of Census tract (c=1,…,n), y_{ic} is term birth weight for tract c, $\mu(y_{ic})$ is the

expected value of the target variable (y_{ic}), tr($\mu(y_{ic})$) is the transformation (e.g. log, box-cox) of $\mu(y_{ic})$, x_p^c is the concentration averages or their transformation of the pth air pollutant during a certain trimester or the pregnancy, a_{0c} is the intercept, β_c^p is the regular or transformed (e.g. log) health effect (birth weight per unit increase in exposure) of the pth air pollutant; other confounders include non-linear ones (x_{jc}) such as NDVI and maternal age, as well as factor variables x_{kc} such as race/ethnicity, diabetes, hypertension and preeclampsia. $\mathbf{s}_c$() is the semi-parametric spline function and f_c() is the factor function. $\mu(y_{ic})$ is the expected value of the ith individual y_{ic} conditional on their neighborhood $E(y_{ic} \mid \text{All } \mu(y_{lc(l \neq i)}))$. y_{ic}, a_{0c} and β_{pc} are assumed to be normally distributed: $y_{ic} \sim N(\mu_c, \sigma_c)$, a_{0c} or $\beta_{pc} \sim N(0, \sigma_p)$.

$\varepsilon^c \sim N(0, \Sigma^c)$, $\Sigma^c = [\sigma_{ij}^c]$ represents spatial autocorrelation (σ_{ij}^c between the ith and j^{th} locations) that is incorporated into the model as spatial effects. ε^c for individual k can be expressed as:

$$\varepsilon_k^c = \rho \sum_{h \neq k} w_{hk} \, (y_{ic} - \mu(y_{ic})) \quad (2)$$

The Matérn covariance function can be used to determine the range (r_C) and nugget (n_C) of spatial weight matrix [1]:

$$C(d) = \sigma^2 \frac{1}{\Gamma(v)2^{v-1}} (\sqrt{2v}\frac{d}{p})^v K_v(\sqrt{2v}\frac{d}{p}) \quad (3)$$

where $C(d)$ is the Matérn covariance (variogram) function and the distance, d between h and k. Γ is the gamma function, K_v is the modified Bessel function of the second kind, , and ρ and v are non-negative parameters of the covariance:

$$w_{hk} = \begin{cases} 0 & \text{if } d_{hk} \geq r_C \\ \frac{1}{n_C + d_{hk}} & \text{otherwise} \end{cases} \quad (4)$$

In this study, Moran's I [2] was used to test spatial autocorrelation; if spatial autocorrelation is statistically significant and it can be incorporated into the model [3].

The effect of air pollutant and the parameters of confounders were calculated based on the

posterior distribution using full Bayesian inference:

$$p_c\left(a_{0c}, \beta_c^p, a_f, s, \tau^2, b \middle| y\right) \propto p_c\left(y \middle| a_{0c}, \beta_c^p, a_f, s, \tau^2, b\right) p_c\left(a_{0c}, \beta_c^p, a_f, s, \tau^2, b\right)$$

$$= \prod_{i=1}^{n_c} L_i(\beta_c^i; \eta_c^i) \prod_{j=1}^{m_c} \{p(s_c^j | \tau_{cj}^2) p(\tau_{cj}^2)\} \prod_{g=1}^{G_c} \{p(b_C^g | v_{cg}^2) p(v_{cg}^2)\} \quad (5)$$

where n_c is the total number of the samples within the census tract, c, a_{0c} is the intercept, β_c^p is the health effect of the pth air pollutant, a_f is the differential intercepts for the factor variables, b represents the random effects, s is the spline functions for the non-linear confounders, the likelihood $L_i(\beta_c^i; \eta_c^i)$ is determined by the distribution of β_c^i and the predictors, η_k^i (i is the sample index), $p(\tau_{cj}^2)$ is the prior distribution of the variance τ_{cj}^2 for the spline function, $p(v_{cg}^2)$ is the prior distribution of the variance, v_{cg}^2 for random effects. Bayesian inference via Markov Chain Monte Carlo (MCMC) simulation is based on updating full conditionals of single parameters or blocks of parameters [4].

Stage Two: the effects (β) of air pollutants on term birth weight were modeled against the tract-level covariates to examine spatial variability of β across tracts and the effects of the tract-level modifiers for β.

$$\begin{cases} \beta_p \sim N_k(m(\beta_p), V_p) \\ m(\beta_p) = h(\eta_p) \\ \eta_p = \alpha_0 + \sum_{j=1}^{m_p} s(c_j) + \varepsilon_p \\ \mu(\beta_p) = E(\beta_p \mid Nei(\beta_{pj(j \neq c)})) \end{cases} \quad (6)$$

where p represents the pth pollutant (NO_2 or NO_x), $m(\beta_p)$ represents expected estimates for $\hat{\beta}_p$, $h(\eta_p)$ is the link function for $\mu(\beta_p)$ ($h(\eta_p) = \eta_p$ for normal distribution), c_j is the socio-demographic, exposure-related and land-use factor at the tract level, $s(c_j)$ is the semi-parametric non-linear spline function for the factor c_j. The intercept, a_0 represents the average or background estimate of air pollution effect [$a_0 \sim N(0, \sigma_p)$]. ε_p is assumed to be spatial auto-correlative ($\varepsilon_p | \Sigma \sim N_p(0, \Sigma^p)$) and spatially conditional auto-correlative regression (CAR) was used to model it ($\Sigma^p = [\sigma_{ij}^p]$ represents spatial covariance) [1]. The variance of β_p measures the variability of air pollution effects across the census tracts.

The conditional expectation of the target variable (β_p) is used to represent spatial effects [4] and determined by the tract-level factors at the location, c and a weighted sum of the mean-centered residuals at neighborhood [$Nei(\beta_p^{j \neq c})$ in equation (6)]. The residual to incorporate spatial correlation influence from neighborhood is:

$$\varepsilon_p^c = \rho \sum_{j \neq c} w_{cj} \, (\beta_p^j - \mu_p^c) \qquad (7)$$

where ρ represents effects of spatial neighborhood to be estimated, w_{cj} is spatial weight determining the relative influence of neighborhood location j on location c [1] (w_{cj}=1 if tract c is a neighbor of tract j, 0 otherwise).

Similar to equation (5), point estimates of the posterior effects of air pollutant and non-linear associations of the tract-level factors with the effects were calculated based on the posterior distribution using full Bayesian inference. Bayesian inference via MCMC simulation is based on updating full conditionals of single parameters or blocks of parameters given the test and data [4].

For the first stage, no spatial effect was incorporated in the model because of insignificant spatial autocorrelation for 91% census tracts according to the test of Moran's I. The health effect of NO_2 and NO_x in each Census tract was estimated using JAGS (a program of Bayesian hierarchical models using MCMC simulation; run in R by the interface package, rjags). For the second stage, significant spatial autocorrelation was observed according to the test of Moran's I (p-value<0.05) and we used BayesX to establish the Bayesian hierarchical models with the incorporation of spatial effects (equation 2). JAGS was used in stage one because JAGS but not BayeX supports the inclusion of informative priors (e.g. the mean and variance of the prior effect of NO_2, summarized from the previous studies, Table S1) for the fixed effects of air pollutants. BayesX was used in the second stage since it is more efficient to incorporate spatial effects within additive models than JAGS [5]. In BayesX, uninformative priors were used for the intercept and spatial term was automatically imposed as smooth term with the sum-to-zero constraint on the errors [6]. We include the major codes for the two stage models as additional files 2-5 (NO2: stage_one_no2.R, stage_two_no2.R; NOx: stage_one_nox.R, stage_two_nox.R).

Table S1 Effects of NO_2 and NO_x on birth weight from the previous studies

Type	Region	Pollutant	Study size	Results (95%CI)	Reference
Study for specific region	US	NO_2	400,000	-1.24 g (-18.9g to 16.42 g) for per ppb increase	Bobak & Leon [7]
	Seoul, S. Korea	NO_2	276,763	-1.83 g for per ppb increase	Ha et al. [8]
	Sao Paulo, Brazil	NO_2	179,460	- 7.0 g (-14.3 g to 0.3 g) for 10 μg/m^3 increase	Gouveia et al. [9]
	California	NO_2	3,901	-7.2 g (-34.7 g to 20.4 g) for IQR (25 ppb) increase	Salam et al. [10]
	Brisbane	NO_2	28,200	102.9 g (-70.0 g to 275.7 g) for per 20 ppb increase	Hansen et al. [11]
	Connecticut	NO_2	358,504	-8.9 g (-10.8 g to -7.0 g) for IQR (4.8 ppb) increase	Bell et al. [12]
	Sabadell, Spain	NO_2	570	34.8 g (- 94.4 g to 164.0 g) for per 20 ppb increase	Aguilera et al. [13]
	England/Wales	NO_2	56,525	-81.0 g (-102.8 g to -59.2 g) for per 20 ppb increase	Jackson et al. [14]
	Oslo, Norway	NO_2	25,229	-19.8 g (-62.8 g to 23.2 g) for per 20 ppb increase	Madsen et al. [15]
	California	NO_2	3,545,177	- 18.0 g (-19.2 g to -16.8 g) for 20 ppb increase	Morello-Frosch et al. [16]
	Valencia	NO_2	787	-36.6 g (-125.0 g to 51.8 g) for 20 ppb increase	Ballester et al. [17]
	Poitiers, Nancy	NO_2	776	-41.4 g (-150.5 g to 67.7 g) for 20 ppb increase	Lepeule et al. [18]
	Dutch cities	NO_2	3,853	18.8 g (-47.4 g to 85 g) for per 20 ppb increase	Parker et al. [19]
	Atlanta	NO_2	406,627	- 4.5 g (-8.5 g to - 0.6 g) for per quartile increase (IQR: 5.0 ppb)	Darrow et al. [20]
Review	(Review)[a]	NO_2	Variance:	-28.1 g (-44.8 g to 11.5 g) for 20 ppb increase	Stieb et al. [21]

Abbreviations: CI, Confidence interval; IQR: Inter Quartile Range; AOR, adjusted odds ratio; g: gram; US: United States.

[a](Review): not specific-region but from the review paper based on the studies of multiple regions

Table S2 Contribution of each tract-level factor in linear models to the NO_2 and NO_x effects

Covariate (unit)	NO_2	NO_x
Distance to the freeways/highways (km)	4.1e-5 (-7.4e-6,9.5e-5)[a]	1.8E-4 (7.1e-5, 2.8e-4)
Proportion of taking vehicles to work #	-0.61 (-1.30, 0.11)	-3.29 (-5.65, -1.02)
Proportion of taking bikes or walk to work	0.43 (-0.65, 1.54)	4.96 (1.50, 8.34)
Proportion of travel time to work<30m	#	0.44 (-1.79,2.71)
Proportion of White race	0.10 (-0.35,0.54)	#
Proportion of Black race	-0.19 (-1.11,0.76)	-0.04 (-1.94, 1.91)
Proportion of Asian race	-0.26 (-1.10, 0.56)	
Proportion of women with no education#	-0.86 (-2.89, -1.19)	#
Proportion of women with bachelor plus education #	0.05 (-2.13, 2.24)	#
Proportion of gas use for heating	-0.71 (-1.17, -0.24)	-2.21 (-3.67,-0.61)
Proportion of electricity power facility land-use	-10.75 (-20.23,-1.04)	-15.41 (-43.03,-12.45)
Median family income (USD)	1.17e-6 (-1.99e-6, 4.13e-6)	2.8e-5 (3.4e-7, 5.6e05)
Proportion of heavy industry land-use	-3.85 (-7.36,-0.06)	-0.86 (-1.22, -0.54)
Proportion of park and recreational land-use	#	15.40 (7.81, 22.89)
Mean NDVI	2.17 (1.25, 3.10)	#

#: Result not shown due to statistical insignificance.

[a]. effect coefficient in linear models (mean effect with 95% confident intervals for each change)

Table S3. Change in effects of NO_2 and NO_x between the 1st and 4th quartiles of each tract-level factor in non-linear models

Covariate (unit)	Intervals of range (min-max)	NO_2		NO_x	
		Effect change (g per 10 ppb)[a]	Percent for average effect	Effect change (g per 10 ppb)[a]	Percent for average effect
Average posterior effect of air pollutants (reference)[a]		-14.7	1	-6.9	1
Distance from freeways/highways (km)[b]	0-40	3.7	25%[a]	4.9	71%
Percent of population driving vehicles to work #	0-92%	-1.6	-10%	-14.7	-213%
Percent of population taking bikes or walk to work	0-50%	1.2	8%	6.5	94%
Percent of population commuting to work < 30 minutes	33-95%	-	-	5.9	86%
Percent of Whites population	2-75%	0.3	2%	-	-
Percent of Blacks population	6-92%	-3.9	-27%	-3.7	-53%
Percent of Asian population	0-60%	-2.3	-16%	-	-
Percent of women with no or lower education level below bachelor#	0-50%	-5.1	-34%	-	-
Percent of women with bachelor or higher educational level #	0-50%	5.2	35%	-	-
Percent of population using gas for heating	7-97%	-1.8	-12%	-17.4	-252%
Percent of electrical power facility land-use	0-12%	-31.4	-213%	-62.3	-902%
Median household income (USD)	7271-200064	1.0	7%	3.4	49%
Percent of heavy-industry land-use	0-32%	-13.0	-88%	-3.9	-56%
Percent of park and recreational land-use	0-35%	-	-	6.7	97%
Mean NDVI	0-0.5	4.8	33%	-	-

-: Result not shown due to no statistical significance. [a] negative sign indicates adverse effect (from the regression models), i.e. decrease of term birth weight by NO_2 and NO_x for the total or average posterior effect. [b]: change in effects of air pollutants between the 1st and 4th quartiles of the tract-level factors, with the posterior average effects as the reference, positive value indicating decrease in adverse effects while negative value indicating increase in adverse effects

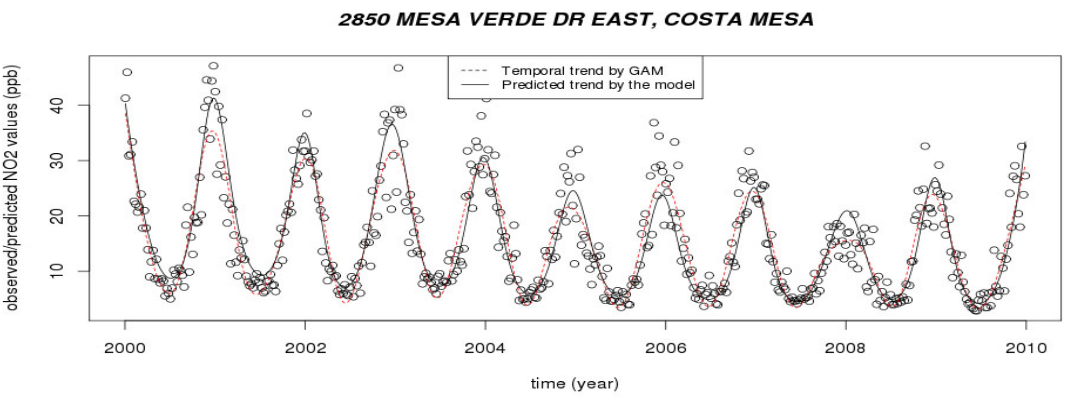

a. NO_2

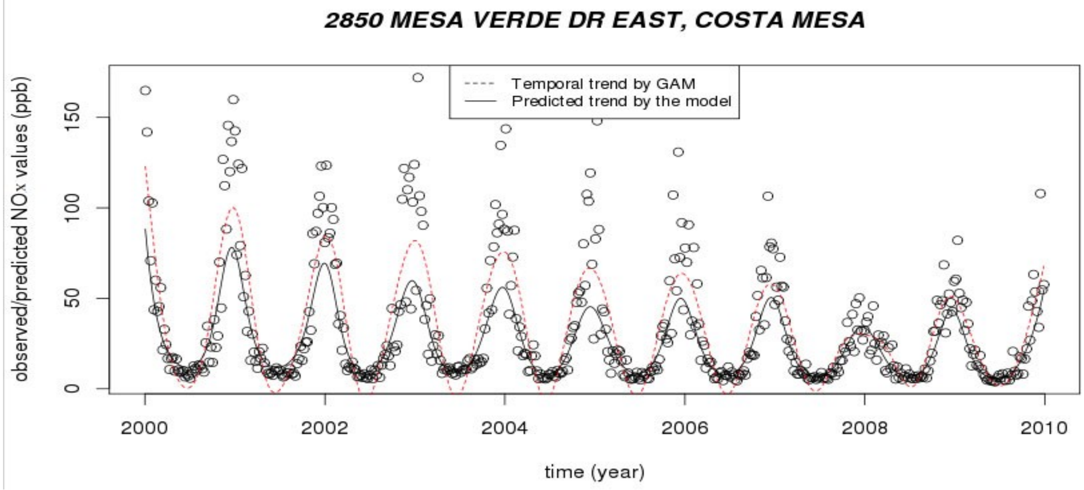

b. NO_x

Figure S1 NO_2 and NO_x time series predicted by the spatiotemporal model

(Station address: 2850 Mesa Verde Dr. East, Costa Mesa)

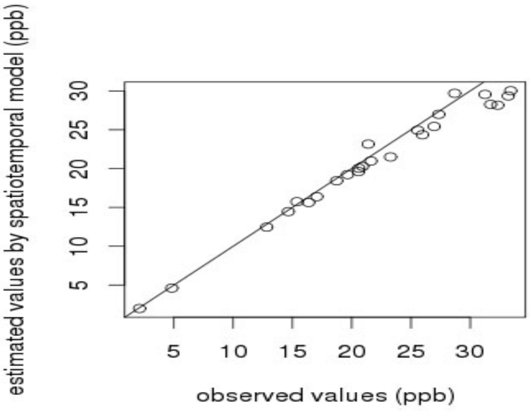

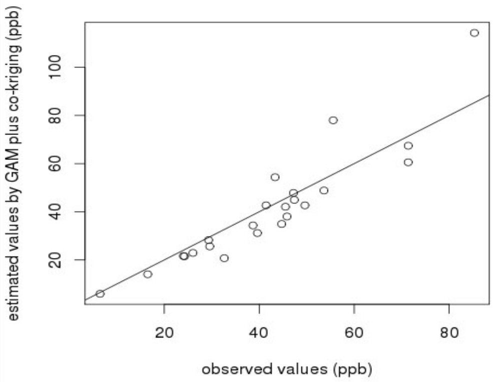

a. NO_2 b. NO_x

Figure S2 NO_2 (a) and NO_x (b) long-term averages of the predicted time series vs. the measurements for all the 25 stations

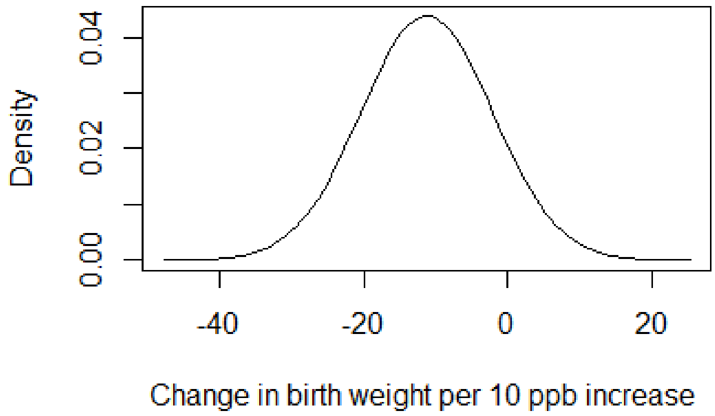

μ=-11.2 g per 10 ppb change, σ^2=82.9

Figure S3. *A priori* statistics of the effects of NO_2 on birth weight summarized from the previous studies

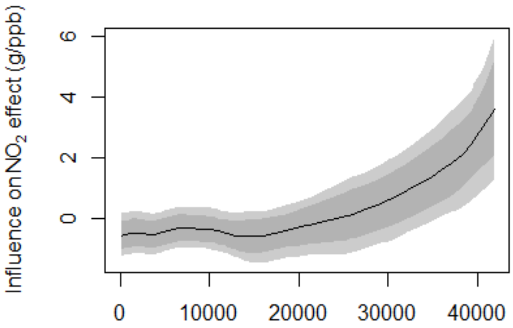

a. Distance from freeways/highways (m)

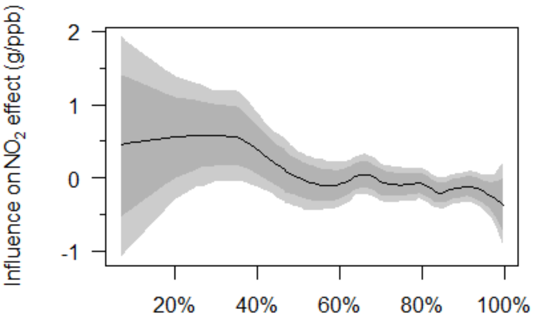

b. Percent of population driving to work

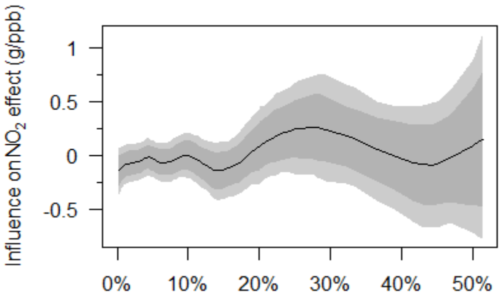

c. Percent of population who bike/walk to work

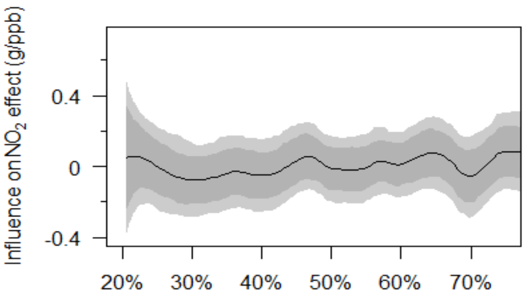

d. Percent of Whites

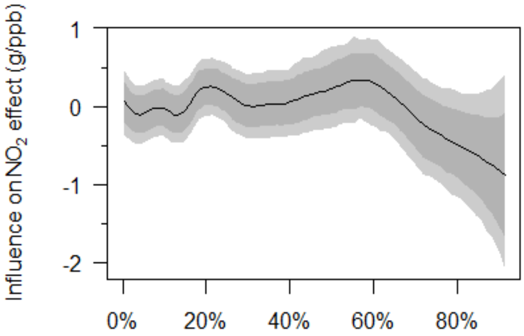

e. Percent of Blacks

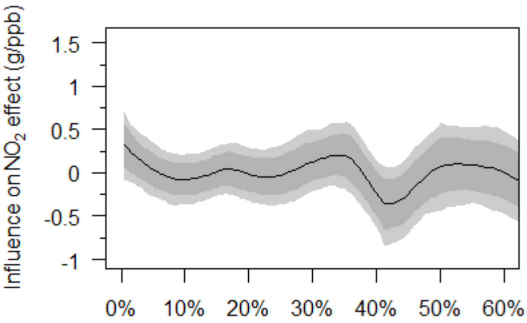

f. Percent of Asians

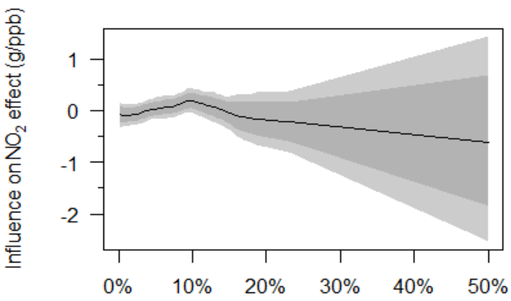

g. Percent of women with no or low education

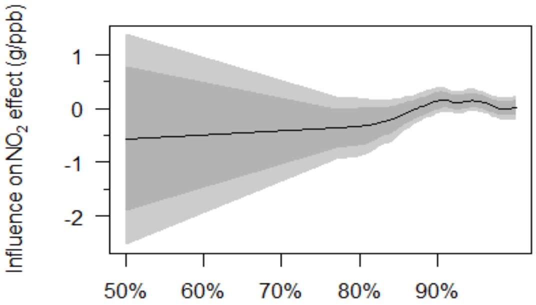

h. Percent of women with bachelor or higher education

Figure S4. Non-linear effects of NO2 on birth weight (smooth term) from stage two (to be continued)

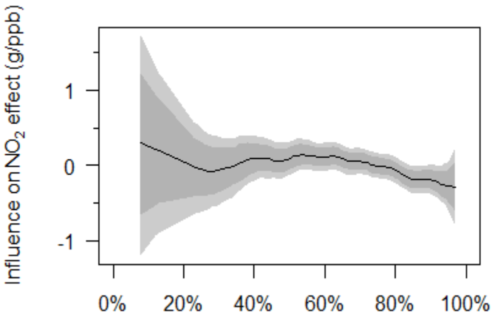

i. Percent of population who use gas for heating

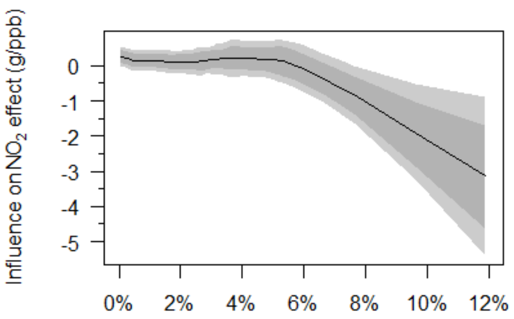

j. Percent of electricity power facility land-use

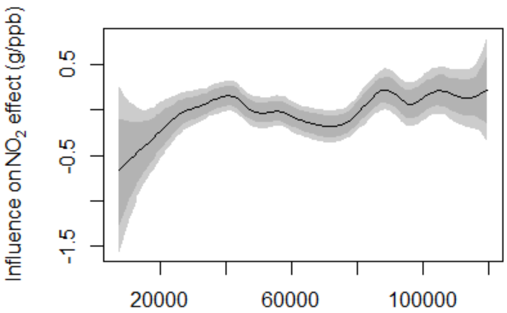

k. Median household income

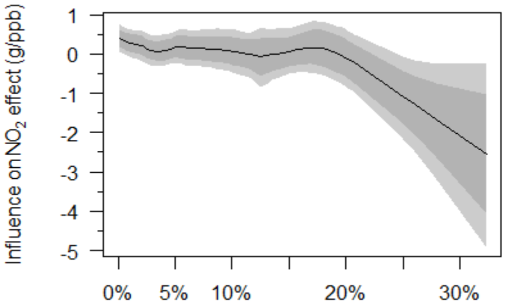

l. Percent of heavy industry land-use

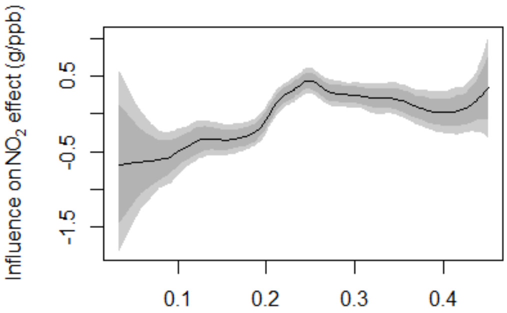

m. Mean NDVI

Figure S4. Non-linear effects of NO_2 on birth weight (smooth term) from stage two (continued)

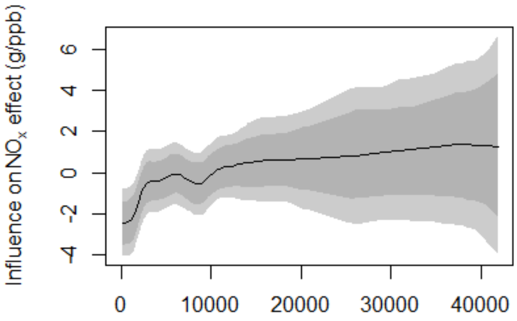

a. Distance from freeways/highways (m)

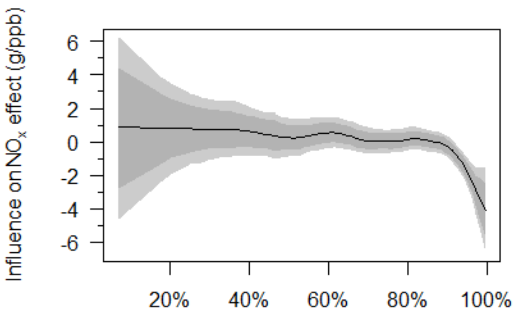

b. Percent of population driving to work

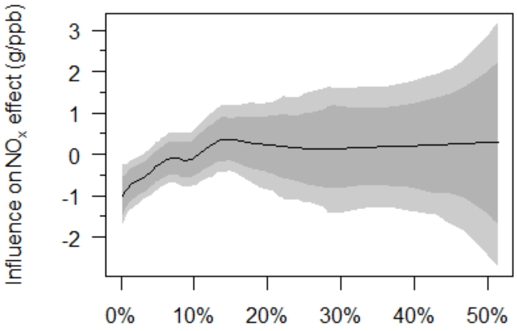

c. Percent of population who bike or walk to work

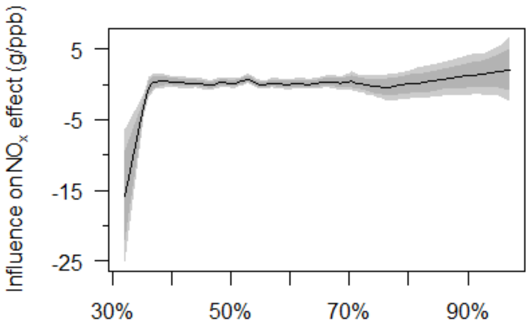

d. Percent of population with commuting time < 30 m

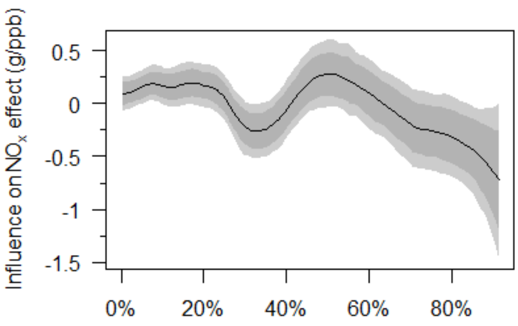

e. Percent of Blacks

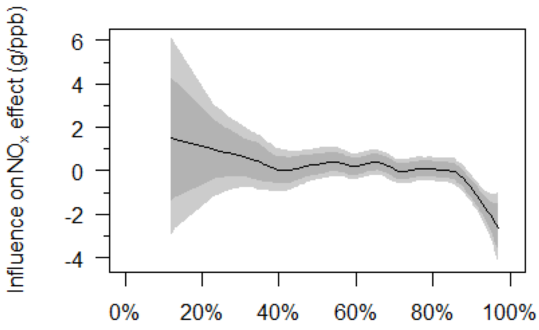

f. Percent of population who use gas for heating

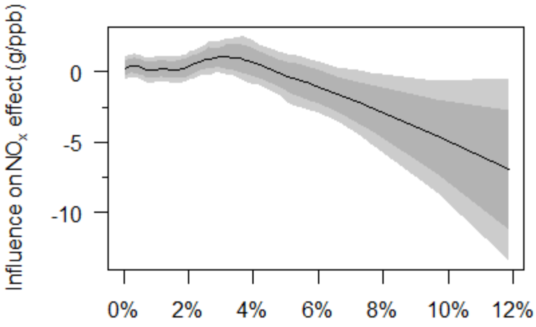

g. Percent of electricity power facility land-use

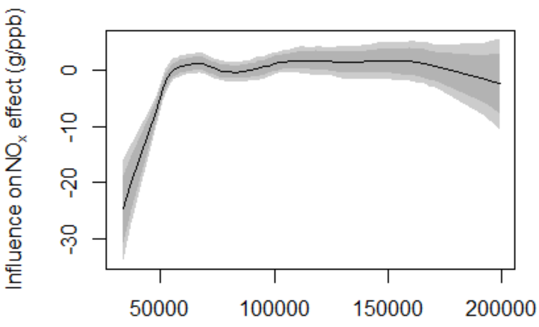

h. Median household income

Figure S5. Non-linear effects of NO_x on birth weight (smooth term) from stage two (to be continued)

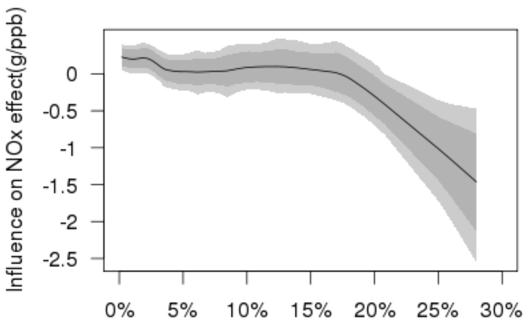

i. Percent of heavy industry land-use

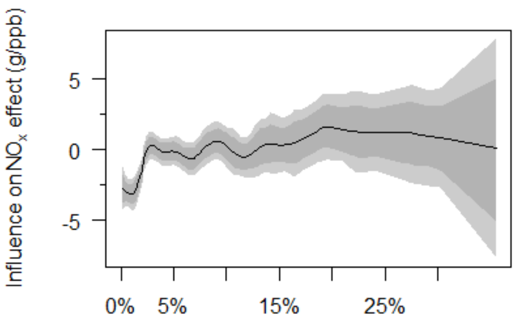

j. Percent of park and recreational land-use

Figure S5. Non-linear effects of NO_x on birth weight (smooth term) from stage two (continued)

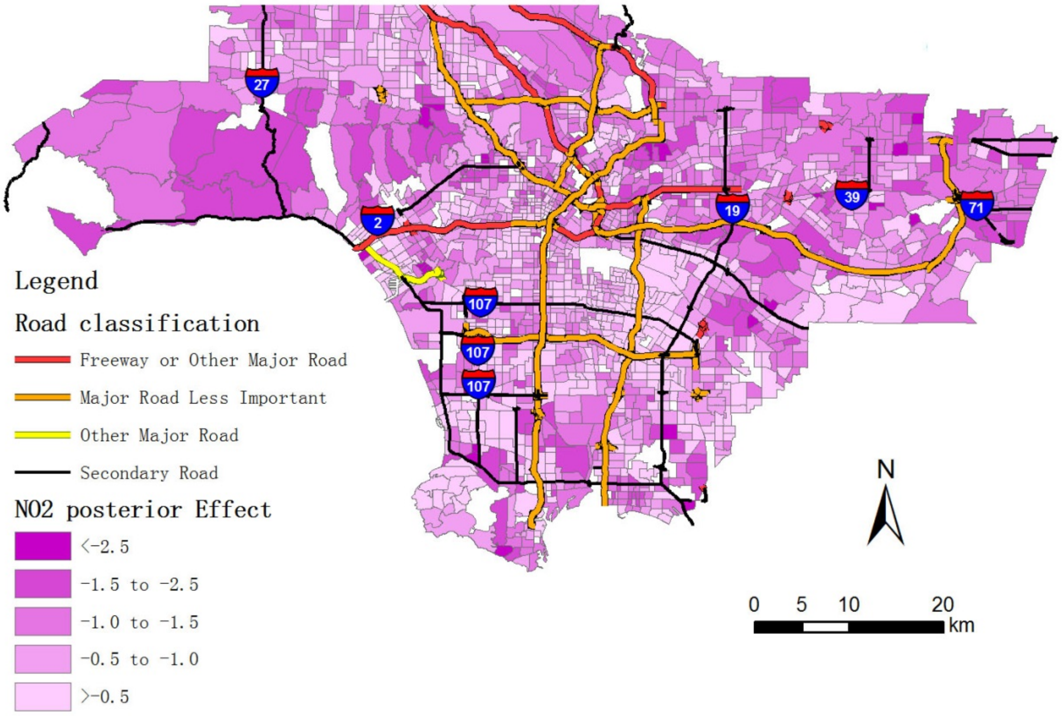

a. NO_2

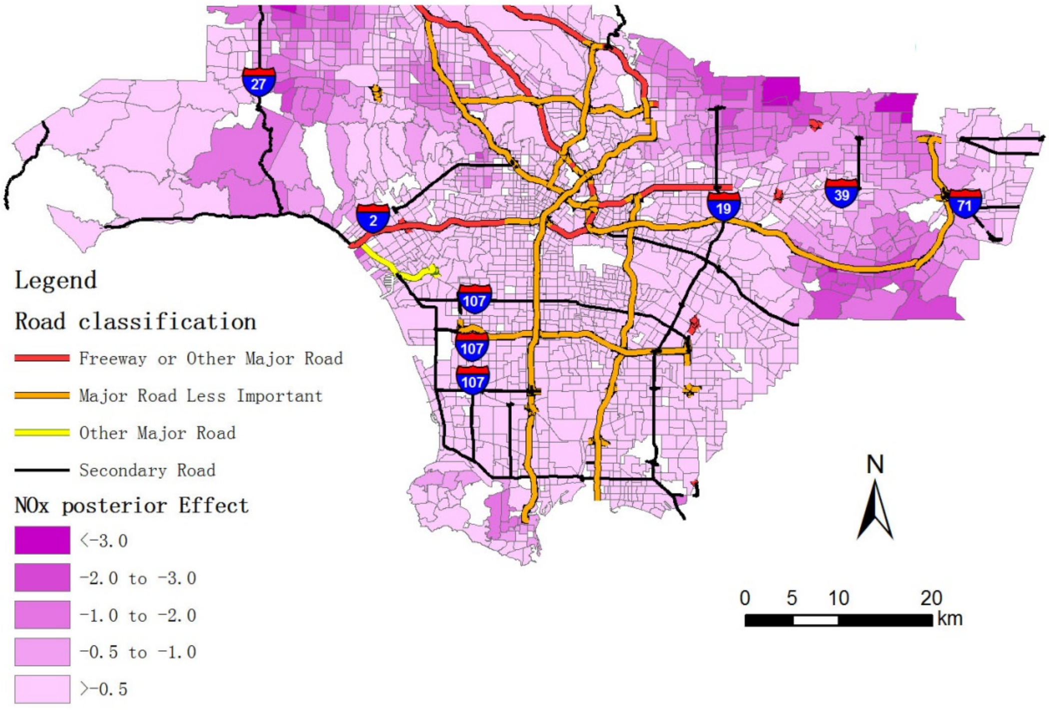

b. NO_x

Figure S6. Posterior estimates of the effects of NO_2 and NO_x

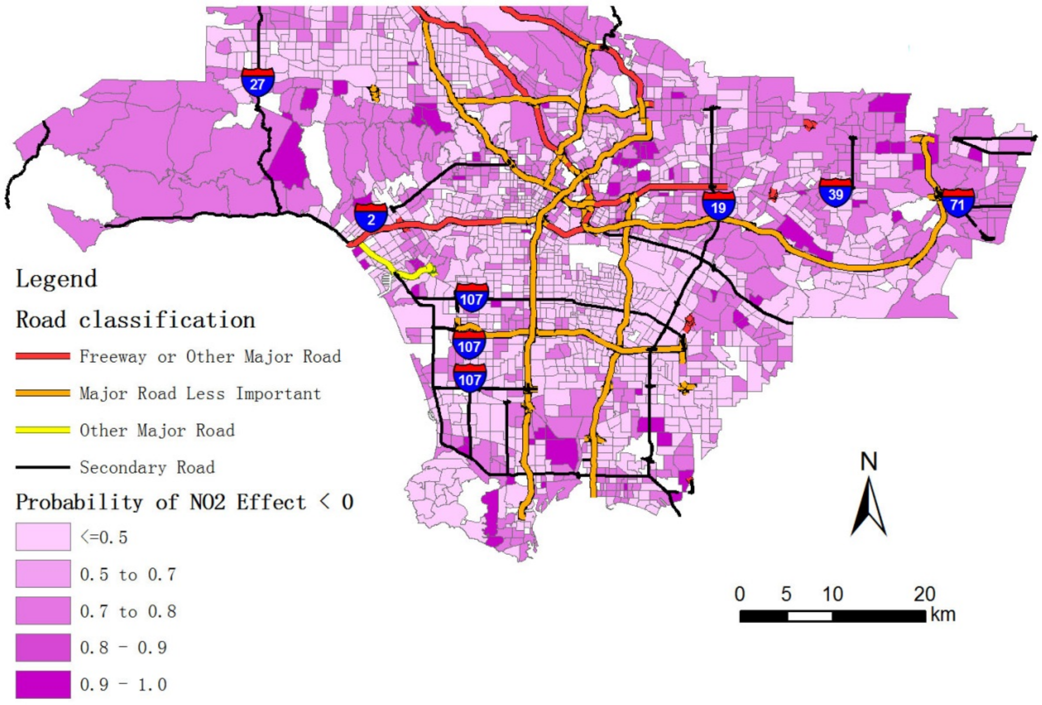

a. NO_2

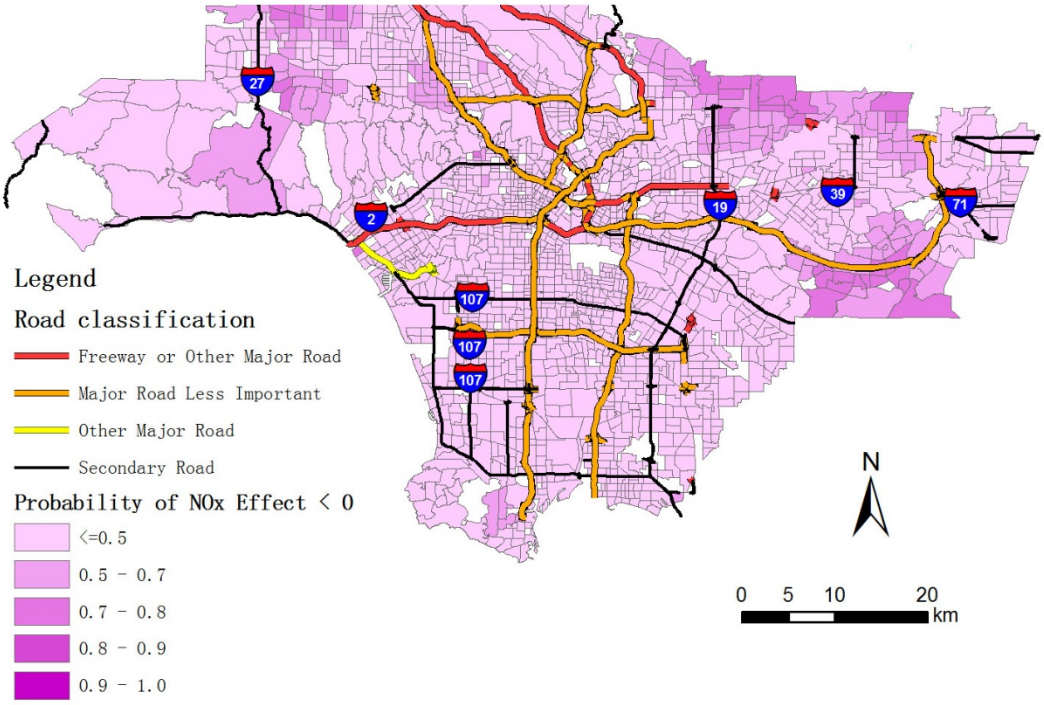

b. NO_x

Figure S7. Probability map of Census tract NO_2 and NO_x effects for term birth weight [$P(\beta < 0)$]

References

1. Cressie N. Statistics for Spatial Data, Revised Edition. New York: Wiley; 1993.

2. Li HF, Calder CA, Cressie N. Beyond Moran's I: Testing for spatial dependence based on the spatial autoregressive model. Geogr Anal. 2007;39(4):357-75.

3. Dormann CF. Response to comment on "Methods to account for spatial autocorrelation in the analysis of species distributional data: a review". Ecography. 2009;32(3):379-81.

4. Fahrmeir L, Lang S. Bayesian inference for generalized additive mixed models based on Markov random field priors. J Roy Stat Soc C-App. 2001;50:201-20.

5. Belitz C, Brezger A, Klein N, Kneib T, Lang S, Umlauf N. BayesX: Methodology Manual2015.

6. Kandala N, Ghilagaber G. Advanced Techniques for Modelling Maternal and Child Health in Africa. Demographic Methods and Population Analysis. New York: Springer; 2014.

7. Bobak M, Leon DA. Pregnancy outcomes and outdoor air pollution: an ecological study in districts of the Czech Republic 1986-8. J Occup Environ Med. 1999;56(8):539-43.

8. Ha EH, Hong YC, Lee BE, Woo BH, Schwartz J, Christiani DC. Is air pollution a risk factor for low birth weight in Seoul? Epidemiology. 2001;12(6):643-8.

9. Gouveia N, Bremner SA, Novaes HMD. Association between ambient air pollution and birth weight in Sao Paulo, Brazil. J Epidemiol Commun H. 2004;58(1):11-7.

10. Salam MT, Millstein J, Li YF, Lurmann FW, Margolis HG, Gilliland FD. Birth outcomes and prenatal exposure to ozone, carbon monoxide, and particulate matter: results from the Children's Health Study. Environ Health Perspect. 2005;113(11):1638-44.

11. Hansen C, Neller A, Williams G, Simpson R. Maternal exposure to low levels of ambient air pollution and preterm birth in Brisbane, Australia. BJOG. 2006;113(8):935-41.

12. Bell ML, Ebisu K, Belanger K. Ambient air pollution and low birth weight in Connecticut and Massachusetts. Environ Health Perspect. 2007;115(7):1118-24.

13. Aguilera I, Guxens M, Garcia-Esteban R, Corbella T, Nieuwenhuijsen MJ, Foradada CM et al. Association between GIS-based exposure to urban air pollution during pregnancy and birth weight in the INMA sabadell cohort. Environ Health Perspect. 2009;117(8):1322-7.

14. Jackson CH, Best NG, Richardson S. Bayesian graphical models for regression on multiple data sets with different variables. Biostatistics. 2009;10(2):335-51.

15. Madsen C, Gehring U, Walker SE, Brunekreef B, Stigum H, Naess O et al. Ambient air pollution exposure, residential mobility and term birth weight in Oslo, Norway. Environ Res. 2010;110(4):363-71.

16. Morello-Frosch R, Jesdale BM, Sadd JL, Pastor M. Ambient air pollution exposure and full-term birth weight in California. Environ Health. 2010;9:44.

17. Ballester F, Estarlich M, Iniguez C, Llop S, Ramon R, Esplugues A et al. Air pollution exposure during

pregnancy and reduced birth size: a prospective birth cohort study in Valencia, Spain. Environ Health. 2010;9:6.

18. Lepeule J, Caini F, Bottagisi S, Galineau J, Hulin A, Marquis N et al. Maternal exposure to nitrogen dioxide during pregnancy and offspring birth weight: comparison of two exposure models. Environ Health Perspect. 2010;118(10):1483-9.
19. Parker JD, Rich DQ, Glinianaia SV, Leem JH, Wartenberg D, Bell ML et al. The international collaboration on air pollution and pregnancy outcomes: initial results. Environ Health Perspect. 2011;119(7):1023-8.
20. Darrow LA, Klein M, Strickland MJ, Mulholland JA, Tolbert PE. Ambient Air Pollution and Birth Weight in Full-Term Infants in Atlanta, 1994-2004. Environ Health Perspect. 2011;119(5):731-7.
21. Stieb DM, Chen L, Eshoul M, Judek S. Ambient air pollution, birth weight and preterm birth: A systematic review and meta-analysis. Environ Res. 2012;117:100-11.
